# The Great Recession and inequalities in access to health care: a study of unemployment and unmet medical need in Europe in the economic crisis

**DOI:** 10.1093/ije/dyx193

**Published:** 2017-09-18

**Authors:** Joana Madureira-Lima, Aaron Reeves, Amy Clair, David Stuckler

**Affiliations:** 1 Department of Sociology, University of Oxford, Oxford, UK; 2 International Inequalities Institute, London School of Economics and Political Science, London, UK

**Keywords:** Health inequalities, unemployment, access to healthcare, out-of-pocket-payments, recession

## Abstract

**Background:**

Unmet medical need (UMN) had been declining steadily across Europe until the 2008 Recession, a period characterized by rising unemployment. We examined whether becoming unemployed increased the risk of UMN during the Great Recession and whether the extent of out-of-pocket payments (OOP) for health care and income replacement for the unemployed (IRU) moderated this relationship.

**Methods:**

We used the European Survey on Income and Living Conditions (EU-SILC) to construct a pseudo-panel (*n* = 135 529) across 25 countries to estimate the relationship between unemployment and UMN. We estimated linear probability models, using a baseline of employed people with no UMN, to test whether this relationship is mediated by financial hardship and moderated by levels of OOP and IRU.

**Results:**

Job loss increased the risk of UMN [β = 0.027, 95% confidence interval (CI) 0.022–0.033] and financial hardship exacerbated this effect. Fewer people experiencing job loss lost access to health care in countries where OOPs were low or in countries where IRU is high. The results are robust to different model specifications.

**Conclusions:**

Unemployment does not necessarily compromise access to health care. Rather, access is jeopardized by diminishing financial resources that accompany job loss. Lower OOPs or higher IRU protect against loss of access, but they cannot guarantee it. Policy solutions should secure financial protection for the unemployed so that resources do not have to be diverted from health.

## Introduction

The declining trend in unmet medical need (UMN) in Europe suffered a reversal at the onset of the Great Recession in 2008 as more people began reporting that they were foregoing care because it was too expensive.^1^^,^^2^ In response to the crisis some governments increased co-payments for some services and treatments, but these changes were implemented some time after the crisis, suggesting that this initial rise may be explained by declining incomes.

**Key Messages**

- Job loss decreased access to medical care during the Great Recession in the European Union.
- Financial hardship is one of the mechanisms through which unemployment affects access to medical care. Unemployment, thus, does not necessarily compromise access to health care. Rather, access is jeopardized by diminishing financial resources that accompany job loss.
- Fewer people experiencing job loss lost access to medical care in countries where out-of-pocket payments wse low and where income replacement is high.
- Policy solutions should secure financial protection for the unemployed so that resources do not have to be diverted away from health.

Incomes fall due to unemployment and, in many European economies, the unemployed may have limited access to healthcare. Reductions in incomes occur even in countries where unemployment insurance and severance packages are generous.^3^^,^^4^ When incomes fall, families are sometimes faced with tough decisions; for example if health is poor, some households may prioritize spending on housing or food before health expenditure.^5–7^ Savings and severance packages may temporarily enable households to smooth consumption, even though earnings have fallen.

Financial strain, then, may mediate the relationship between becoming unemployed and the inability to access health care.^8^ In other words, the loss of income due to unemployment will erode whatever financial buffer a household may have had and subsequently affect health care consumption.

National health and social protection policies may break this association by decreasing or removing financial barriers to access to medical care and providing income replacement for the unemployed (IRU). Many countries, however, require co-payments when accessing health care, and higher co-payments have been shown to impair health care-seeking behaviour.^9^^,^^10^ In these contexts, citizens often pay more for their health care ‘out-of-pocket’ and so the impact of job loss UMN may be concentrated in countries where out-of-pocket payments (OOP) for health care are higher or where IRU is lower.

Rising UMN may save governments money in the short term because fewer people may be utilizing state-financed or -subsidized health care. There are, however, medium- and long-term health concerns. Delays in seeking diagnosis and treatment may lead to worse health outcomes,^11^ and they may result in preventable hospitalizations in the medium term, which may actually increase net costs.^12^

Preliminary research indicates that loss of access to the health system is concentrated among those on low incomes, the elderly and the unemployed.^1^^,^^15^ Naturally, addressing changes in UMN will require accounting for any health selection into unemployment. There is a longstanding literature exploring whether people with poor health are more likely to lose their job than people with good health.^13–16^ Moreover, the healthy worker effect may change during recessions, suggesting that people in poor health may be more likely to lose work.^17^ Accordingly, we test the hypothesis of both a confounding and a mediating effect of health status in the association between becoming unemployed and the probability of reporting UMN.

In this paper, using a uniquely constructed dataset, we test whether: (i) becoming unemployed increases the probability of reporting UMN; (ii) the association is mediated by the reduced availability of individual resources; and (iii) the association between becoming unemployed and the probability of reporting UMN is moderated by OOP spending on health care as well as by the level of IRU.

## Methods

We collected EU-SILC data between 2008 and 2010 because: (i) the recession began in 2008, leading to widespread unemployment in Europe (EUROSTAT 2015); and (ii) preliminary empirical analysis of the UMN trend indicates sudden and substantial reversal of the decline in UMN in 2008.^1^ We follow these individuals until 2010, because in 2011 EU governments started enacting austerity measures that affected unemployment, incomes, OOP and access to health care, thereby potentially changing the relationship between job loss and UMN.^16–18^

### Building the dataset

The EU SILC releases a cross-sectional and a longitudinal version. Some of the data captured in the former are not in the latter and vice versa. This was the case with variable of interest: UMN. This limits our ability to explore how changing circumstances for any particular individual may be associated with changes in UMN. Therefore, in order to optimize the research design possibilities offered by the employment status data collected yearly from the same individual in the longitudinal arm and the access to health care data in the cross-sectional arm, we produced a pseudo-panel for UMN. We used a matching procedure to identify observations that had the same characteristics in the two arms of the survey according to a set of predetermined variables. Observations that were not matched were removed from the analytical data. Matching percentages reached figures of 95% of individuals in the longitudinal arm in the best years and 88% in the least good. The composition of the matched sample is representative of the survey population, suggesting that non-matched observations were effectively missing-at-random.

To assess the impact of a transition to unemployment, we restricted the sample to those interviewed in 2009 and 2010 and who in the previous year were employed and did not report UMN, allowing us to capture exclusively the hypothesized direction of change from employment into unemployment, making the most of the panel structure of the data. The restrictions resulted in a sample size of 135 529 observations. Country sample sizes varied between 1480 in Finland to 14 751 in Italy.

### The outcome variable

UMN was measured using the question: ‘Was there any time during the past 12 months when you really needed to consult with a doctor, undertake medical examination or medical treatment but did not?’ ‘Yes, at least one occasion’ was coded ‘1’ and ‘No, no occasion’ was coded ‘0’). Need for dental care was ascertained in a separate question.^19^

### Predictor and control variables

The predictor variable, ‘employment status’ has four response categories: ‘Employed’, ‘Unemployed’, ‘Retired’ and ‘Inactive’. SILC collects this variable as a self-declared current ‘main activity status’, determined on the basis of the most time spent on the activity in question, but no criteria have been specified. ‘If a person has lost a job, then the situation as of the time of the interview should be reported. In this sense, ‘current’ overrides any concept of averaging over any specific reference period’.^19^ We restricted the analysis to the first two categories, to capture transitions for people active in the labour market.

Standard sociodemographic controls were included in the analyses: age, a quadratic term for age, sex, education, marital status, country and year of survey. Income quintile is a categorical variable constructed from the variable ‘Total disposable household income’. The 1st quintile represents the top 20% earners and the 5th, the lowest. ‘Difficulty to Make Ends Meet’ is a binary variable created from grouping the five categories of the original variable: ‘With great difficulty’, ‘With difficulty’ and ‘With some difficulty’ were grouped into ‘Difficult to Make Ends Meet’ and ‘Fairly easily’ and ‘Easily’. ‘Self-Rated Health’ that was ‘Very Good’ and ‘Good’ was grouped into ‘Good Health’ (the reference category); ‘Fair Health’ remained coded as such, and ‘Very Bad’ and ‘Bad’ were grouped into ‘Bad Health’.

The variable OOP made by households as a percentage of total health expenditure was collected for each country and year from the WHO Health for All database. The variable IRU was created from expenditure data on yearly country unemployment benefits in euros, divided by the number of unemployed, both obtained from Eurostat. This proxy measure gives an overview of government commitment to spending on unemployment support, but cannot measure the generosity of support at the individual level.

### Statistical models

We used random (RE), fixed effects (FE) and linear probability models (LPM)^20^ as well as country random coefficients multilevel models. RE models are represented by Equations 1 and 2.

#### 

##### Eq.1 RE model, mediator role of financial strain:

UMNit=µ0+β1Employmentit+ β2Ageit+ β3Age2it+ β4  L. SelfRated Health + Zi+ β5Income Quintileit + β6Ends Meetit+ β7Employmentit * Ends Meetit+ αi+ ɛit

Where i = 1,…, n individuals each of whom is measured at three points t = 1,…, 3 in time. µ_0_ is an intercept. The set of predictor variables that vary over time is represented by the name of the variable and the subscript _it_. Those that do not vary over time–e.g. Sex, Country of Residence–are represented by the vector Z_i_. The two error terms are represented by α_i_–the combined effect on the dependent variable of all unobserved variables that are constant over timeand ɛ_it_–the purely random variation at each point in time.

To test whether poor health confounds the association, we introduced a lagged variable capturing individual health in the year preceding unemployment. Restrictions to the sample dictate that only individuals who in the preceding year were employed and had their health needs met were analysed. If they had poor health in the previous year and were also employed, it is unlikely that the cause for dismissal was poor health. To test poor health’s mediating effect, ideally we would have used a lead variable for self-rated health to account for the possibility that ill health followed unemployment. However, the restrictions imposed on the sample yielded too few observations. Instead, we tested the hypothesis using self-rated health in the year of job loss.

Additionally, we restricted the sample to those whose health status had not changed, and used both RE and FE LPMs. The former addresses the possibility that those who are chronically ill lose their jobs more easily and also have more UMN than those who are not, by, within the unchanged health group, controlling for health status. We tested the mediating effect of financial hardship by controlling for Income and Difficulty to Make Ends Meet, and by adding an interaction term between the two. As a robustness check we used an FE LPM as well as random coefficient between and within effects linear probability. Moreover, we applied the initial LPM to a restricted sample of those gaining employment

##### Eq.2 RE model, moderator role of OOP and IRU:

UMNit=µ0+ β1Employmentit+ β2Ageit+ β3Age2it + zi+ β4SelfRated Health + β5Income Quintileit + β6Ends Meetit+ β7IRU/ β7OOP αi+ ɛit

where z_i_ is a vector of time invariant variables. IRU is a binary control variable denoting the country-level measure above or below the median level of IRU, and OOP a binary control variable of the country-level measure above or below the median level of OOP.

Last, we tested whether two country-level variables (1.OOP and 2.IRU) may moderate the association between job loss and reporting UMN. We split both variables by their median values and then re-estimate the association between job loss and reporting UMN within each set of countries (Eq.2). First we look at OOP, comparing countries with low spending (≤ 19.78%) with countries with high spending (> 19.78%). Next we examine whether the association between job loss and reporting UMN varies according to the income replacement rate for unemployed persons, comparing countries with a low level of IRU (≤ 7081.794 euroes per year and countries with IRU (> 7081.794 euroes per year). Additionally, we calculated predicted probabilities of UMN at different employment statuses by type of OOP country.

## Results

The percentage of those transitioning from employment into unemployment in the study period is 4.02%; 4.69% transitioned from having all of their health needs met into UMN (Table 1).

**Table 1:** Sample characteristics

		2008	2009	2010
Unmet health need	No unmet need	125316 (92.52%)	125818 (93%)	126002 (93.07%)
	Unmet need	10133 (7.48%)	9469 (7%)	9384 (6.93%)
Economic activity	Employed	85938 (55.75%%)	83223 (53.98%)	81918 (53.15%)
	Unemployed	6540 (4.24%)	8558 (5.55%)	9366 (6.1%)
	Retired	34092 (22.11%)	36188 (23.47%)	37927 (16.99%)
	Other inactive	27589 (17.9%)	26201 (16.99%)	24903 (16.15%)
Gender	Male	73629 (47.7%)	73629 (47.7%)	73629 (47.7%)
	Female	80751 (52.3%)	80751 (52.3%)	80751 (52.3%)
Age	Mean age	47.52	48.52	49.52
Marital status	Married	92541 (60.29%)	92595 (60.36%)	92735 (60.44%)
	Never married	40497 (26.38%)	39678 (25.86%)	38899 (25.35%)
	No longer married	20457 (13.33%)	21136 (13.78%)	21808 (14.21%)
Income quintiles	1st quintile	33592	28149	30851
	2nd quintile	31165	31445	29979
	3rd quintile	29849	32022	30722
	4th quintile	30161	31299	31128
	5th quintile	29539	31391	31660
Education level	Primary only	19629 (13%)	19396 (12.80%)	19274 (12.72%)
	Secondary only	101056 (66.89%)	100714 (66.50%)	99724 (65.82%)
	Post-secondary	30391 (20.01%)	31358 (20.7%)	32523 (21.46%)
Ability to make ends meet	Difficult to make ends meet	91367 (59.3%)	92636 (60.1%)	93571 (60.7%)
	Not difficult to make ends meet	62709 (40.7%)	61513 (39.9%)	60556 (39.3%)
Self-rated health	Good health	87258 (64.77%)	85939 (64.09%)	85215 (63.61%)
	Fair health	33215 (24.66%)	33628 (25.08%)	33785 (25.22%)
	Poor health	14244 (10.57%)	14534 (10.84%)	14969 (11.17%)
OOP as % of total health expenditure		19.15%	18.90%	19.00%
Sample countries:	Austria, Belgium, Bulgaria, Cyprus, Czech Republic, Denmark, Estonia, Greece, Spain, Finland, France, Hungary, Iceland, Italy, Lithuania, Luxembourg, Latvia, Malta, The Netherlands, Norway, Poland, Portugal, Romania, Sweden, Slovenia, Slovakia, UK			

### Unemployment, unmet medical need and the confounding role of health status

The coefficient for the simple bivariate association between unemployment and UMN is β = 0.027 [95% confidence interval (CI) 0.022–0.033: *P* < 0.001), or, if 1000 people lost their jobs, then approximately 27 would also lose access to health care. In Spain, for example, where the number of unemployed went from 5.013 million in 2011 to 5.811 million in 2012 (EUROSTAT 2015), our model predicts that roughly 21 546 people may have experienced UMN due rising unemployment.

After controlling for demographic factors, those who become unemployed are 1.4% more likely to experience UMN than those who remain employed (95% CI 0.008–0.019: *P* < 0.001). Health status does not confound or mediate the association, as the probability remains fairly stable after the introduction of lagged and current health status at 1.2% (95% CI 0.007–0.018: *P* < 0.001) and 1.1% (95% CI 0.006–0.017: *P* < 0.001), respectively (Table 2).

**Table 2:** Linear probability of UMN on employment status, controlling for sociodemographic and health status

	Population facing unmet medical need			
	(1)	(2)	(3)	(4)
Employment status (baseline = employed)				
Unemployed	0.027^**^	0.014^**^	0.012^**^	0.011^**^
	[0.022–0.033]	[0.0080–0.019]	[0.0067–0.018]	[0.0055–0.017]
Sex (baseline = male)				
Female		0.0013	0.00057	−0.00034
		[−0.0019–0.0045]	[−0.0026–0.0038]	[−0.0035–0.0028]
Age		0.0031^**^	0.0031^**^	0.0029^**^
		[0.0021–0.0041]	[0.0020–0.0041]	[0.0019–0.0039]
Age sq		−0.000030^**^	−0.000031^**^	−0.000033^**^
		[−0.000041–0.000018]	[−0.000043–0.000020]	[−0.000044–0.000022]
Education (baseline = primary education)				
Secondary and other non-tertiary		−0.014^**^	−0.013^**^	−0.0094^**^
		[−0.021–0.0076]	[−0.019–0.0057]	[−0.016–0.0026]
Tertiary		−0.025^**^	−0.021^**^	−0.016^**^
		[−0.032–0.018]	[−0.029–0.014]	[−0.023–0.0091]
Marital status (baseline = married)				
Never married		−0.00068	−0.00098	−0.0020
		[−0.0048–0.0034]	[−0.0051–0.0031]	[−0.0061–0.0021]
No longer married		0.015^**^	0.014^**^	0.013^**^
		[0.010–0.020]	[0.0090–0.019]	[0.0081–0.018]
Lagged self rated health (baseline = good)				
1l: fair health			0.021^**^	
			[0.018–0.025]	
2l: bad health			0.036^**^	
			[0.029–0.044]	
Self-rated health (baseline = good)				
Fair health				0.045^**^
				[0.042–0.049]
Bad health				0.078^**^ [0.070–0.085]
Country and year dummies	—	—	—	—
Constant	0.055^**^	−0.028^*^	−0.027^*^	0
	[0.053–0.057]	[−0.052–0.0026]	[−0.052–0.0020]	[0–0]
Observations	127665	127242	125902	125632

95% confidence intervals in brackets. Sample restricted to those who in the previous year were employed and had their health needs met.

*P < 0.05, **P < 0.01.

Next, we restrict our sample to those whose health has not changed during the study period, and still find that those who lost their jobs are 0.087% (95% CI 0.003–0.015: *P* < 0.001) more likely to report UMN than those who remained employed. Those in bad health, however, are more likely to report UMN (Table 1A, available as Supplementary data at *IJE* online) than those in good health. The FE model applied to this restricted sample confirms these findings: job loss increased the probability of UMN by 1.5% (95% CI 0.006–0.025: *P* < 0.001) among those in poor health (Table 2A, available as Supplementary data at *IJE* online).

To address the possibility that reporting UMN was a result of a malaise associated with unemployment rather than a real clinical need, we used an FE regression of self-rated health on UMN on a baseline of individuals who were in good or fair health and had no UMN. Individuals falling into UMN show an increase in the probability of reporting worse health (Table 3A, available as Supplementary data at *IJE* online).

### The mediating role of financial hardship

Controlling for income and financial hardship attenuates the main coefficient from β = 0.012 (95% CI 0.007–0.018: *P* < 0.001) to 0.0089 (95% CI 0.003–0.015: *P* = 0.002), in support of the mediating effect hypothesis. Further support comes from the interaction between unemployment and the ability to make ends meet. The unemployed who lose the ability to make ends meet show a 2.5% increase in probability of reporting UMN vs those who maintain it. Unemployment per se does not directly cause decreased access to the health system (Table 3). Rather, the loss of financial buffering that comes with unemployment seems to drive the loss of access.

**Table 3:** Linear probability of UMN on employment status: mediating and moderating effect of financial hardship

	Population facing unmet medical need			
	(1)	(2)	(3)	(4)
Unemployed	0.012^**^	0.010^**^	0.0089^**^	−0.011
	[0.0067–0.018]	[0.0047–0.016]	[0.0032–0.015]	[−0.024–0.0021]
Sex (baseline = male)				
Female	0.00057	0.00057	0.00042	0.00045
	[−0.0026–0.0038]	[−0.0026–0.0038]	[−0.0028–0.0036]	[−0.0027–0.0036]
Age	0.0031^**^	0.0028^**^	0.0028^**^	0.0027^**^
	[0.0020–0.0041]	[0.0018–0.0038]	[0.0017–0.0038]	[0.0017–0.0038]
Age sq	−0.000031^**^	−0.000029^**^	−0.000028^**^	−0.000028^**^
	[−0.000043–0.000020]	[−0.000040–0.000018]	[−0.000039–0.000016]	[−0.000039–0.000016]
Education (baseline = primary education)				
Secondary and other non- tertiary education	−0.013^**^	−0.0097^**^	−0.0085^*^	−0.0085^*^
	[−0.019–0.0057]	[−0.017–0.0029]	[−0.015–0.0017]	[−0.015–0.0016]
tertiary education	−0.021^**^	−0.016^**^	−0.012^**^	−0.012^**^
	[−0.029–0.014]	[−0.023–0.0082]	[−0.020–0.0052]	[−0.020–0.0051]
Marital status (baseline = married)				
Never married	−0.00098	−0.0032	−0.0032	−0.0032
	[−0.0051–0.0031]	[−0.0074–0.00089]	[−0.0074–0.00089]	[−0.0074–0.00089]
No longer married	0.014^**^	0.0097^**^	0.0092^**^	0.0091^**^
	[0.0090–0.019]	[0.0046–0.015]	[0.0040–0.014]	[0.0040–0.014]
Self-rated health (baseline = good health)				
1l: fair health	0.021^**^	0.021^**^	0.020^**^	0.020^**^
	[0.018–0.025]	[0.017–0.024]	[0.017–0.023]	[0.017–0.023]
2l: bad health	0.036^**^	0.035^**^	0.034^**^	0.034^**^
	[0.029–0.044]	[0.027–0.042]	[0.026–0.041]	[0.026–0.041]
Income (baseline = 5th income quintile)				
4th quintile		0.0073^**^	0.0058^**^	0.0058^**^
		[0.0035–0.011]	[0.0020–0.0096]	[0.0021–0.0096]
3rd quintile		0.014^**^	0.012^**^	0.012^**^
		[0.0096–0.019]	[0.0072–0.016]	[0.0073–0.016]
2nd quintile		0.019^**^	0.016^**^	0.016^**^
		[0.014–0.025]	[0.011–0.022]	[0.011–0.022]
1st quintile		0.034^**^	0.029^**^	0.029^**^
		[0.027–0.040]	[0.023–0.036]	[0.023–0.036]
Not difficult make ends meet			0	0
			[0–0]	[0–0]
Difficult make ends meet			0.013^**^	0.012^**^
			[0.010–0.016]	[0.0096–0.015]
Baseline unemployed: not difficult to make ends meet				
Unemployed: difficult to make ends meet				0.024^**^ [0.010–0.039]
Country and year dummies	—	—	—	—
Constant	−0.027^*^	−0.030^*^	−0.034^**^	−0.034^**^
	[−0.052–0.0020]	[−0.055–0.0048]	[−0.059–0.0093]	[−0.059–0.0088]
Observations	125902	125869	125797	125797

95% confidence intervals in brackets. Sample restricted to those who in the previous year were employed and had their health needs met.

*P < 0.05; **P < 0.001.

Again, we re-estimate our models adjusting for person-specific differences that are constant over time, finding that those who lose their jobs and cannot make ends meet are 2.9% more likely to report UMN than those who can (Table 4A, available as Supplementary data at *IJE* online). As a validity check, we estimated the effect of gaining employment, controlling for demographic and economic factors, which shows a protective effect β = −0.026 (95% CI -0.086–0.033) (Table 5A, available as Supplementary data at *IJE* online).

The initial RE model assumed that the error term was not correlated with the predictors, which in turn, allowed time-invariant variables to play a role as explanatory variables. We addressed this assumption by estimating a country-level random coefficients model with between and within effects (Table 6A, available as Supplementary data at *IJE* online). The coefficient for the effect of unemployment on UMN varies according to unobserved country-level characteristics, potentially related to the welfare state. The within country coefficient (β = 0.010, 95% CI 0.001–l0.019: *P* = 0.025) is positive, similar to that obtained using random effects (β = 0.0089). The between country effect is larger in countries where the unemployment rate rises one unit above the average proportion of unemployed: the probability of UMN increases by 31% (β = 0.31, 95% CI 0.030–0.59: *P* = 0.030).

### The moderating role of out-of-pocket payments and income replacement

**Figure 1:**
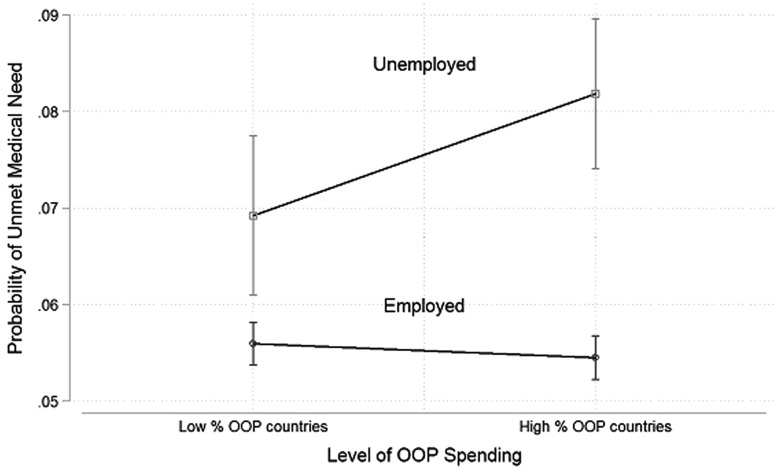
Predicted probabilities of UMN according to OOP type. Notes: Vertical lines are 95% confidence intervals.

The effect of becoming unemployed on the probability of reporting UMN is smaller in low-OOP countries, with a 1.6% increase (β = 0.016 (95% CI 0.0080–0.023: *P* = < 0.001), than in high-OOP countries, with a 2.5% increase (β = 0.025 (95% CI 0.017–0.033: *P* < 0.001), controlling for hardship and IRU (Table 4). However, there is no clear difference in relationship between job loss and UMN in low-IRU countries (β = 0.022, 95% CI 0.014–0.030: *P* < 0.001) and high-IRU countries (β = 0.019, 95% CI 0.010–0.027: *P* < 0.001), controlling for hardship and OOP (Table 5). Additionally, we calculated predicted probabilities of reporting UMN in the two types of OOP countries (Figure 1).

**Table 4:** Linear probability regression of unmet health need on employment status in low- and high-OOP countries

	Low-OOP countries	High-OOP countries
Employment status (baseline = employed)		
Unemployed	0.016^**^	0.025^**^
	[0.0080–0.023]	[0.017–0.033]
Sex (baseline = male)		
Female	−0.0038	0.0040
	[−0.0078–0.00025]	[−0.00073–0.0088]
Age	0.0017^*^	0.0030^**^
	[0.00038–0.0030]	[0.0015–0.0045]
Age sq	−0.000016^*^	−0.000027^**^
	[−0.000030–0.0000014]	[−0.000043–0.0000100]
Education (baseline = primary education)		
Secondary and other non-tertiary education	−0.0097^*^	−0.0015
	[−0.019–0.00076]	[−0.011–0.0075]
Tertiary education	−0.010^*^	−0.0067
	[−0.019–0.00065]	[−0.017–0.0033]
Marital status (baseline = married)		
Never married	−0.00018	−0.0055
	[−0.0052–0.0048]	[−0.012–0.00098]
No longer married	0.0011	0.013^**^
	[−0.0051–0.0073]	[0.0054–0.021]
Self-rated health (baseline = good)		
Fair health	0.016^**^	0.022^**^
	[0.011–0.020]	[0.018–0.027]
Good health	0.017^**^	0.043^**^
	[0.0069–0.027]	[0.032–0.054]
Income (baseline = 5th income quintile)		
4th income quintile	0.0099^**^	0.0090^*^
	[0.0059–0.014]	[0.0015–0.016]
3rd income quintile	0.019^**^	0.018^**^
	[0.014–0.025]	[0.0097–0.026]
2nd income quintile	0.026^**^	0.026^**^
	[0.019–0.033]	[0.017–0.035]
1st income quintile	0.042^**^	0.037^**^
	[0.034–0.050]	[0.027–0.046]
Financial hardship (baseline = not difficult to make ends meet)		
Difficult to make ends meet	0.012^**^	0.017^**^
	[0.0090–0.016]	[0.013–0.022]
IRU (baseline = low income replacement)	0.043^**^	−0.0050
	[0.037–0.048]	[−0.012–0.0016]
Constant	−0.036^*^	−0.053^**^
	[−0.066–0.0062]	[−0.088–0.019]
Observations	63987	61810

95% confidence intervals in brackets. Sample restricted to those who in the previous year were employed and had their health needs met. Sample split into low- and high-copayment countries.

*P < 0.05; **P < 0.01.

**Table 5:** Linear probability regression of UMN on employment status in low- and high-IRU countrie

	(Low-IRU countries)	(High-IRU countries)
Employment status (baseline = employed)		
Unemployed	0.022^**^	0.019^**^
	[0.014–0.030]	[0.010–0.027]
Sex (baseline = male)		
Female	0.0030	−0.0028
	[−0.0017–0.0078]	[−0.0074–0.0018]
Age	0.0026^**^	0.0023^**^
	[0.0012–0.0041]	[0.00080–0.0038]
Age sq	−0.000022^**^	−0.000023^**^
	[−0.000038–0.0000055]	[−0.000040–0.0000066]
Education (baseline = primary education)		
Secondary and other non-tertiary education	−0.000087	−0.013^**^
	[−0.0099–0.0097]	[−0.022–0.0044]
Tertiary education	−0.0020	−0.014^**^
	[−0.013–0.0086]	[−0.023–0.0042]
Marital status (baseline = married)		
Never married	−0.0035	−0.0040
	[−0.010–0.0030]	[−0.0096–0.0015]
No longer married	0.011^**^	−0.00066
	[0.0037–0.018]	[−0.0081–0.0068]
Self-rated health (baseline = good)		
Fair health	0.024^**^	0.011^**^
	[0.020–0.029]	[0.0070–0.016]
Bad health	0.036^**^	0.023^**^
	[0.025–0.046]	[0.012–0.034]
Income (baseline = 5th income quintile		
4th income quintile	0.0039	0.011^**^
	[−0.0058–0.014]	[0.0069–0.015]
3rd income quintile	0.015^**^	0.023^**^
	[0.0054–0.025]	[0.018–0.028]
2nd income quintile	0.028^**^	0.031^**^
	[0.018–0.038]	[0.022–0.039]
1st income quintile	0.046^**^	0.047^**^
	[0.036–0.056]	[0.034–0.060]
Financial hardship (baseline = Not difficult to make ends meet)		
Difficult to make ends meet	0.014^**^	0.015^**^
	[0.0093–0.018]	[0.011–0.019]
OOP (baseline = Low−OOP countries)	0.0083^**^	−0.0062^**^
	[0.0042–0.013]	[−0.011–0.0017]
Constant	−0.061^**^	−0.0023
	[−0.096–0.026]	[−0.036–0.031]
Observations	63412	62385

95% confidence intervals in brackets. Sample restricted to those who in the previous year were employed and had their health needs met. Sample split into low- and high-income replacement countries.

*P < 0.05; **P < 0.01.

As a robustness check, we used an FE model on the split samples and obtained similar results (Tables 7A and 8A, available as Supplementary data at *IJE* online). What is more, the effects on UMN of the individual falling from good health into fair and ill health are over three times larger in high-OOP countries that in their low-OOP counterparts, which suggests that as the individual’s health deteriorates and need is accumulated, the capacity to meet that need is reduced in high-OOP countries. Individuals who become unemployed in low-IRU countries show a 1.1% increased probability of UMN (β = 0.011, [95% CI -0.00089–0.023: *P* > 0.05), whereas those in high-IRU countries show a 2.2% probability (β = 0.022, 95% CI 0.0093–0.035: *P* < 0.001). It is worth noting, however, that low-IRU individuals in bad health, and who face financial hardship, are six and four times more likely to report UNM, respectively, than their counterparts in high-IRU countries.

## Discussion

We find an association between becoming unemployed and increased UMN. This association is not explained by health selection; in fact, it is positive even for those individuals whose health remained stable before and after job loss. Whereas we exclude the possibility that a change in health status motivates job loss, there is still room for the possibility that those who are chronically ill lose their jobs more easily and also have more UMN than those who are not. We find, however, that even when we control for health status, our results remain constant.

When household OOPs are higher, the association between job loss and UMN is stronger, suggesting that more people becoming unemployed lose access to health care in these contexts. Likewise, when IRU is lower, the association between job loss and UMN is stronger than when IRUs are more generous.

This relationship will create and exacerbate health inequalities as it penalizes the unemployed, those facing financial strain and those in poorer health. Our results suggest that unemployment is more strongly correlated with UMN in countries with higher OOP. However, we see a slightly different relationship with IRU. Although overall UMN is lower in countries with more generous financial protection for the unemployed, this does not benefit the unemployed more than those in work. Exemptions from co-payments for those claiming unemployment insurance may protect those households, but there may be a sizeable share of the population who are unemployed and not claiming unemployment insurance, who experience higher risk of UMN. Notwithstanding this uncertainty, it is troubling that co-payments appear to exacerbate the impact of job loss by restricting access to care. Such inequities undermine the stated goals of health systems developed by the WHO and the European Commission.^21^^,^^22^

There are several important limitations. Our study does not capture employment security. Those employed on a precarious contract may also lose access to health care because they may have fewer financial resources and may face additional time constraints. More work is needed to explore whether precarious work is associated with access to health care. Last, our national-level measure of OOP overlooks exemptions from co-payments for some households on the grounds of, for example, low incomes. Moreover, it does not disentangle the effects of differential co-payment levels for primary care and emergency services. Future analyses should unpack this issue in more detail.

Our work has important policy implications. Job loss harms health. What is more, health consequences of job loss may be higher in countries where becoming unemployed also leads to UMN. Reducing OOP or increasing levels of IRU may protect those who lose work from some of the negative health consequences of job loss. Yet, across Europe, many countries have increased OOP, even in primary care, so as to reduce government spending. These policies may have short-term benefits for public spending deficits but, when another recession hits, they may restrict access to health care, particularly among economically vulnerable groups. 

## Supplementary Data

Supplementary data are available at *IJE* online.

**Conflict of interest:** None declared.

## Supplementary Material

Supplementary Data
