# Supplementary material for: The Great Recession and inequalities in access to health care: a study of unemployment and unmet medical need in Europe in the economic crisis
Source: Int J Epidemiol. 2017 Sep 18;47(1):58–68. doi: 10.1093/ije/dyx193 (PMC5837221; doi:10.1093/ije/dyx193)
Supplement: Supplementary Data [file dyx193_ije-2016-09-1127-file003.docx]

Supplementary File for Online Publication

Table 1A. Linear Probability of UMN on Employment Status - Sample restricted to those whose health has not changed

| Population facing unmet medical need | | | | | |
| --- | --- | --- | --- | --- | --- |
|  | (1) | (2) | (3) | (4) | (5) |
| *Employment Status (baseline =Employed)* |  |  |  |  |  |
| Unemployed | 0.0087^**^ [0.0027,0.015] | 0.0073^*^ [0.0013,0.013] | 0.0062^*^ [0.00017,0.012] | 0.0051 [-0.00092,0.011] | -0.0082 [-0.022,0.0051] |
| *Sex (baseline=Male)* |  |  |  |  |  |
| Female | 0.00098 [-0.0022,0.0042] | -0.00065 [-0.0039,0.0025] | -0.00062 [-0.0038,0.0026] | -0.00067 [-0.0039,0.0025] | -0.00065 [-0.0038,0.0025] |
| Age | 0.0022^**^ [0.0012,0.0032] | 0.0022^**^ [0.0012,0.0032] | 0.0020^**^ [0.0010,0.0031] | 0.0020^**^ [0.00097,0.0030] | 0.0020^**^ [0.00097,0.0030] |
| ageSq | -0.000021^**^ [-0.000032,-0.0000093] | -0.000026^**^ [-0.000037,-0.000015] | -0.000025^**^ [-0.000036,-0.000013] | -0.000023^**^ [-0.000035,-0.000012] | -0.000023^**^ [-0.000035,-0.000012] |
| Education (baseline=Primary Education) |  |  |  |  |  |
| Secondary and other non Tertiary | -0.015^**^ [-0.022,-0.0084] | -0.011^**^ [-0.018,-0.0036] | -0.0086^*^ [-0.016,-0.0016] | -0.0077^*^ [-0.015,-0.00064] | -0.0077^*^ [-0.015,-0.00063] |
| Tertiary | -0.022^**^ [-0.030,-0.015] | -0.014^**^ [-0.022,-0.0069] | -0.011^**^ [-0.018,-0.0031] | -0.0083^*^ [-0.016,-0.00082] | -0.0083^*^ [-0.016,-0.00081] |
| *Marital Status (baseline=Married)* |  |  |  |  |  |
| Never Married | -0.00011 [-0.0042,0.0040] | -0.0015 [-0.0056,0.0025] | -0.0029 [-0.0070,0.0012] | -0.0028 [-0.0069,0.0013] | -0.0028 [-0.0069,0.0013] |
| No Longer Married | 0.014^**^ [0.0086,0.019] | 0.012^**^ [0.0071,0.017] | 0.0096^**^ [0.0043,0.015] | 0.0091^**^ [0.0038,0.014] | 0.0091^**^ [0.0038,0.014] |
| *Self Rated Health (baseline =Good)* |  |  |  |  |  |
| Fair Health |  | 0.052^**^ [0.047,0.057] | 0.051^**^ [0.046,0.056] | 0.050^**^ [0.045,0.055] | 0.050^**^ [0.045,0.055] |
| Bad Health |  | 0.068^**^ [0.057,0.080] | 0.067^**^ [0.055,0.079] | 0.066^**^ [0.054,0.077] | 0.065^**^ [0.054,0.077] |
| *Income (baseline= 5th Income Quintile)* |  |  |  |  |  |
| 4th Quintile |  |  | 0.0051^**^ [0.0014,0.0088] | 0.0041^*^ [0.00034,0.0078] | 0.0041^*^ [0.00035,0.0078] |
| 3rd Quintile |  |  | 0.0083^**^ [0.0037,0.013] | 0.0065^**^ [0.0019,0.011] | 0.0065^**^ [0.0019,0.011] |
| 2nd Quintile |  |  | 0.012^**^ [0.0063,0.017] | 0.0093^**^ [0.0038,0.015] | 0.0093^**^ [0.0037,0.015] |
| 1st Quintile |  |  | 0.021^**^ [0.014,0.027] | 0.017^**^ [0.011,0.024] | 0.017^**^ [0.011,0.024] |
| *Financial hardship (baseline=Not difficult make ends meet)* |  |  |  |  |  |
| Difficult make ends meet |  |  |  | 0.010^**^ [0.0072,0.013] | 0.0097^**^ [0.0068,0.013] |
| *Baseline =Unemployed # Not difficult make ends meet* |  |  |  |  |  |
| Unemployed # Difficult make ends meet  Country and Year Dummies | --- | --- | --- | --- | 0.017^*^ [0.0018,0.032]  --- |
| Constant | -0.027^*^ [-0.052,-0.0022] | -0.027^*^ [-0.051,-0.0017] | -0.029^*^ [-0.054,-0.0036] | -0.032^*^ [-0.057,-0.0071] | -0.032^*^ [-0.057,-0.0069] |
| Observations | 105708 | 105708 | 105681 | 105622 | 105622 |

95% confidence intervals in brackets ^*^ *p* < 0.05, ^**^ *p* < 0.01

Table 2A. Fixed effects Linear Probability of UMN on Employment Status

| Population facing unmet medical need | | | |
| --- | --- | --- | --- |
|  | (1) | (2) | (3) |
| *Employment Status (baseline=Employed)* |  |  |  |
| Unemployed | 0.015^**^ [0.0057,0.025] | 0.014^**^ [0.0050,0.024] | 0.014^**^ [0.0047,0.024] |
| Age | 0.018^**^ [0.011,0.024] | 0.018^**^ [0.011,0.024] | 0.018^**^ [0.011,0.024] |
| ageSq | 0.00011^**^ [0.000037,0.00018] | 0.00011^**^ [0.000036,0.00018] | 0.00011^**^ [0.000036,0.00018] |
| *Income (baseline=5th Income Quintile)* |  |  |  |
| 4th Quintile |  | 0.0034 [-0.0020,0.0088] | 0.0033 [-0.0021,0.0088] |
| 3rd Quintile |  | 0.0052 [-0.0020,0.012] | 0.0051 [-0.0021,0.012] |
| 2nd Quintile |  | 0.011^*^ [0.0019,0.019] | 0.010^*^ [0.0016,0.019] |
| 1st Quintile |  | 0.020^**^ [0.0090,0.030] | 0.019^**^ [0.0086,0.030] |
| *Financial Hardship (baseline = Not difficult make ends meet)* |  |  |  |
| Difficult make ends meet |  |  | 0.0055^**^ [0.0015,0.0096] |
| Constant | -0.95^**^ [-1.09,-0.81] | -0.95^**^ [-1.09,-0.81] | -0.95^**^ [-1.10,-0.81] |
| Fixed Effects |  |  | yes |
| N | 106046 | 106019 | 105959 |

95% confidence intervals in brackets

Sample restricted to those who in the previous year were employed, had no unmet need and additionally those whose self rated health did not change

^*^ *p* < 0.05, ^**^ *p* < 0.01

Table 3A. Fixed effects linear probability regression of Unmet Health Need on Self Reported Health- Sample restricted to those who at baseline reported good or fair health and no UMN

|  |  |
| --- | --- |
|  | Self-Rated Health |
| *Baseline =No Unmet Health Need* |  |
| Unmet Health Need | 0.050^**^ [0.040,0.061] |
| Constant | 0.31^**^ [0.31,0.31] |
| Fixed Effects |  |
| N | 228888 |

95% confidence intervals in brackets

^*^ *p* < 0.05, ^**^ *p* < 0.01

Table 4A. Linear Probability of UMN on Employment Status

| Population facing unmet medical need | | | | | |
| --- | --- | --- | --- | --- | --- |
|  | (1) | (2) | (3) | (4) | (5) |
| *Employment status (baseline = employed)* |  |  |  |  |  |
| Unemployed | 0.018^**^ [0.0093,0.026] | 0.018^**^ [0.0093,0.027] | 0.017^**^ [0.0082,0.025] | 0.017^**^ [0.0079,0.025] | -0.021^*^ [-0.041,-0.00089] |
| Age | 0.017^**^ [0.011,0.023] | 0.017^**^ [0.011,0.023] | 0.017^**^ [0.011,0.023] | 0.017^**^ [0.011,0.023] | 0.017^**^ [0.011,0.024] |
| ageSq | 0.00019^**^ [0.00012,0.00026] | 0.00019^**^ [0.00012,0.00026] | 0.00019^**^ [0.00012,0.00026] | 0.00019^**^ [0.00012,0.00026] | 0.00019^**^ [0.00012,0.00026] |
| Self-Rated *Health (baseline= Good Health)* |  |  |  |  |  |
| Fair Health |  | 0.020^**^ [0.016,0.025] | 0.020^**^ [0.016,0.025] | 0.020^**^ [0.016,0.025] | 0.020^**^ [0.016,0.025] |
| Bad Health |  | 0.037^**^ [0.027,0.047] | 0.037^**^ [0.027,0.047] | 0.037^**^ [0.026,0.047] | 0.037^**^ [0.027,0.047] |
| *Income (baseline= 5th Income Quintile)* |  |  |  |  |  |
| 4th Income Quintile |  |  | 0.0052 [-0.00017,0.011] | 0.0052 [-0.00021,0.011] | 0.0052 [-0.00022,0.011] |
| 3rd Income Quintile |  |  | 0.0090^*^ [0.0020,0.016] | 0.0090^*^ [0.0019,0.016] | 0.0089^*^ [0.0018,0.016] |
| 2nd Income Quintile |  |  | 0.015^**^ [0.0069,0.024] | 0.015^**^ [0.0067,0.024] | 0.015^**^ [0.0067,0.024] |
| 1st Income Quintile |  |  | 0.027^**^ [0.017,0.037] | 0.027^**^ [0.017,0.037] | 0.026^**^ [0.016,0.037] |
| *Financial hardship (baseline =Not difficult make ends meet)* |  |  |  |  |  |
| Difficult make ends meet |  |  |  | 0.0047^*^ [0.00075,0.0086] | 0.0041^*^ [0.00015,0.0080] |
| *Baseline Unemployed # Not difficult make ends meet* |  |  |  |  |  |
| Unemployed # Difficult make ends meet |  |  |  |  | 0.046^**^ [0.024,0.068] |
| Constant | -1.09^**^ [-1.23,-0.95] | -1.10^**^ [-1.24,-0.95] | -1.10^**^ [-1.25,-0.96] | -1.10^**^ [-1.25,-0.96] | -1.11^**^ [-1.25,-0.96] |
| Fixed Effects |  |  |  |  | yes |
| N | 127665 | 126050 | 126017 | 125945 | 125945 |

95% confidence intervals in brackets. Sample restricted to those who in the previous year had been employed and had their health needs met

^*^ *p* < 0.05, ^**^ *p* < 0.01

Table 5A. Linear Probability of UMN on Gaining Employment

|  | (UMN) | (UMN) |
| --- | --- | --- |
| *Employment Status (baseline =Unemployed)* | -0.034 [-0.093,0.025] | -0.026 [-0.086,0.033] |
| *Sex (baseline=Male)* |  |  |
| Female | 0.030 [-0.028,0.087] | 0.028 [-0.030,0.086] |
| Age | 0.037^**^ [0.019,0.055] | 0.033^**^ [0.015,0.051] |
| ageSq | -0.00041^**^ [-0.00062,-0.00020] | -0.00038^**^ [-0.00060,-0.00017] |
| *Education (baseline =Primary Education)* |  |  |
| Secondary and other non-Tertiary Education | -0.089^*^ [-0.17,-0.0068] | -0.069 [-0.15,0.014] |
| Tertiary Education | -0.026 [-0.15,0.10] | 0.026 [-0.10,0.16] |
| *Marital Status (baseline =Married)* |  |  |
| Never Married | 0.025 [-0.050,0.100] | 0.015 [-0.061,0.091] |
| No Longer Married | 0.051 [-0.027,0.13] | 0.039 [-0.041,0.12] |
| *Self-Rated Health (baseline = Good Health)* |  |  |
| 1L.Fair Health |  | 0.056 [-0.011,0.12] |
| 2L.Bad Health |  | 0.12^**^ [0.038,0.20] |
| *Income (baseline =5th Income Quintile)* |  |  |
| 4th Quintile |  | 0.022 [-0.16,0.20] |
| 3rd Quintile |  | 0.038 [-0.13,0.21] |
| 2nd Quintile |  | -0.023 [-0.20,0.15] |
| 1st Quintile |  | 0.10 [-0.080,0.29] |
| *Financial Hardship (baseline =Not difficult make ends meet)* |  |  |
| Difficult make ends meet |  | 0.028 [-0.095,0.15] |
| Constant | -0.60^*^ [-1.06,-0.14] | -0.60^*^ [-1.09,-0.11] |
| Observations | 1246 | 1231 |

95% confidence intervals in brackets

Sample restricted to those who in the previous year were unemployed and had UMN

^*^ *p* < 0.05, ^**^ *p* < 0

Table 6A Random Coefficient Between and within effects linear probability of Unmet Medical Need on Employment Status

| Population facing unmet medical need | |
| --- | --- |
|  | (1) |
| Unmet medical Need  **Within Effect** |  |
| Sex (baseline = Male) | -0.00025 [-0.0025,0.0020] |
| Age | 0.0023^**^ [0.0016,0.0030] |
| ageSq | -0.000026^**^ [-0.000034,-0.000018] |
| Education | -0.0012 [-0.0034,0.0010] |
| MaritalStatus | 0.0016 [-0.000097,0.0034] |
| EmploymentStatus | 0.010^*^ [0.0013,0.019] |
| SelfRatedHealth | 0.044^**^ [0.042,0.047] |
| Income_Quintile | 0.0043^**^ [0.0029,0.0057] |
| MakeEndsMeet  **Between Effect** | 0.013^**^ [0.010,0.015] |
| Mean Age | -0.042 [-0.11,0.029] |
| Mean ageSq | 0.00044 [-0.00031,0.0012] |
| Mean Education | -0.032 [-0.11,0.043] |
| Mean MaritalStatus | 0.079 [-0.061,0.22] |
| Mean EmploymentStatus | 0.31^*^ [0.030,0.59] |
| Mean SelfRatedHealth | -0.053 [-0.15,0.040] |
| Mean Income_Quintile | 0.019 [-0.00030,0.039] |
| Mean MakeEndsMeet | -0.077 [-0.17,0.015] |
| Constant | 0.93 [-0.59,2.44] |
| Observations | 125529 |

95% confidence intervals in brackets

Sample restricted to those who in the previous year were employed and had their health needs met

^*^ *p* < 0.05, ^**^ *p* < 0.01

Table 7A Fixed effects linear probability regression of Unmet Medical Need on Employment Status in Low and High OOP Countries

| Population facing unmet medical need | | |
| --- | --- | --- |
|  | (Low OOP) | (High OOP) |
| *Employment Status (baseline=Employed)* |  |  |
| Unemployed | 0.015^*^ [0.0029,0.027] | 0.019^**^ [0.0058,0.033] |
| Age | 0.024^**^ [0.016,0.033] | 0.015^**^ [0.0037,0.025] |
| ageSq | 0.000031 [-0.000064,0.00013] | 0.00033^**^ [0.00021,0.00045] |
| *Self-Rated Health*  *(baseline=Good Health)* |  |  |
| Fair Health | 0.010^**^ [0.0046,0.016] | 0.032^**^ [0.025,0.039] |
| Bad Health | 0.018^**^ [0.0056,0.031] | 0.062^**^ [0.044,0.079] |
| *Income (baseline=5th Income Quintile)* |  |  |
| 4th Quintile | 0.0018 [-0.0041,0.0077] | 0.014^*^ [0.0022,0.026] |
| 3rd Quintile | 0.0084^*^ [0.00021,0.017] | 0.015^*^ [0.0012,0.029] |
| 2nd Quintile | 0.011^*^ [0.000053,0.023] | 0.023^**^ [0.0076,0.039] |
| 1st Quintile | 0.031^**^ [0.014,0.047] | 0.031^**^ [0.014,0.048] |
| *Financial Hardship (baseline=Not difficult make ends meet)* |  |  |
| Difficult make ends meet | 0.0035 [-0.0013,0.0084] | 0.0065 [-0.00060,0.014] |
| Constant | -1.11^**^ [-1.30,-0.91] | -1.27^**^ [-1.51,-1.02] |
| Fixed Effects |  |  |
| N | 64208 | 61737 |

95% confidence intervals in brackets

Sample restricted to those who in the previous year were employed and who had no unmet need * p < 0.05, ** p < 0.01

Table 8A Fixed effects linear probability regression of Unmet Health Need on Employment Status in Low and High Income Replacement Countries

|  | (Low IR Countries) | (High IR Countries) |
| --- | --- | --- |
| *Employment Status (baseline=Employed)* |  |  |
| Unemployed | 0.011 [-0.00089,0.023] | 0.022^**^ [0.0093,0.035] |
| Age | 0.015^**^ [0.0050,0.024] | 0.020^**^ [0.011,0.029] |
| ageSq | 0.00027^**^ [0.00017,0.00038] | 0.00010^*^ [0.0000088,0.00020] |
| *Self-Rated Health*  *(baseline=Good Health)* |  |  |
| Fair Health | 0.028^**^ [0.021,0.034] | 0.013^**^ [0.0076,0.019] |
| Bad Health | 0.062^**^ [0.047,0.077] | 0.011 [-0.0026,0.024] |
| *Income (baseline=5th Income Quintile)* |  |  |
| 4th Income Quintile | 0.015^*^ [0.00051,0.029] | 0.0033 [-0.0021,0.0088] |
| 3rd Income Quintile | 0.016^*^ [0.00029,0.032] | 0.0085^*^ [0.00060,0.016] |
| 2nd Income Quintile | 0.023^**^ [0.0067,0.040] | 0.0083 [-0.0038,0.020] |
| 1st Income Quintile | 0.032^**^ [0.014,0.050] | 0.030^**^ [0.013,0.047] |
| *Financial Hardship (baseline=Not difficult make ends meet)* |  |  |
| Difficult make ends meet | 0.0075^*^ [0.0014,0.014] | 0.0020 [-0.0030,0.0069] |
| Constant | -1.18^**^ [-1.40,-0.97] | -1.05^**^ [-1.24,-0.86] |
| Fixed Effects |  |  |
| N | 63327 | 62618 |

95% confidence intervals in brackets

Sample restricted to those who in the previous year were employed and who had no unmet need

^*^ *p* < 0.05, ^**^ *p* < 0.01
